# Surface‐Selective Pt Enrichment for Stabilizing Ultrathin 2D PtCu Nanosheets Toward Durable Oxygen Reduction

**DOI:** 10.1002/advs.78520

**Published:** 2026-10-27

**Authors:** HyunWoo J. Yang, SangJae Lee, KwangHo Lee, HyunWoo Chang, HyunWoo T. Yang, Dongwon Shin, MinJun Kim, EunAe Cho

**Affiliations:** ^1^ Department of Materials Science and Engineering Korea Advanced Institute of Science and Technology (KAIST) Daejeon Republic of Korea

**Keywords:** 2D nanosheets, galvanic replacement reaction, oxygen reduction reaction, shape‐controlled catalysts, surface‐selective Pt enrichment

## Abstract

Ultrathin Pt‐based 2D alloy nanosheets are promising electrocatalysts for the oxygen reduction reaction (ORR) due to their high surface‐to‐volume ratios and favorable facet‐dependent activity. However, their thermodynamic instability and susceptibility to transition metal dissolution present critical barriers to their practical application. While surface Pt enrichment could mitigate such degradation, constructing Pt‐rich surfaces on 2D nanosheets remains challenging, as conventional approaches often induce structural collapse or undesired thickening. Herein, we report a surface‐selective Pt enrichment strategy via galvanic replacement to stabilize ultrathin PtCu nanosheets (PtCu NS/C) while preserving their unique 2D integrity. PtCu@Pt NS/C features a Pt‐enriched outer region and an alloyed subsurface, which effectively suppresses Cu exposure while maintaining beneficial electronic interactions. PtCu@Pt NS/C delivers high ORR activity, outperforming commercial Pt/C. More importantly, PtCu@Pt NS/C exhibits remarkable durability, with 17.5% mass activity loss after 30 000 accelerated stress test cycles. Notably, in single‐cell PEMFC configurations, PtCu@Pt NS/C demonstrates outstanding voltage stability with a minimal loss of 4 mV at 0.2 A cm^−2^, whereas PtCu NS/C and Pt/C suffer much larger losses of 41 and 38 mV, respectively. This work demonstrates that preferential near‐surface Pt enrichment effectively stabilizes PtCu nanosheets, offering a surface‐engineering approach for improving the durability of ultrathin 2D alloy electrocatalysts.

## Graphical abstract

Ultrathin 2D alloy nanosheet catalysts could offer exceptional catalytic activity but suffer from severe structural instability. Here, a surface‐selective Pt enrichment strategy via galvanic replacement effectively stabilizes PtCu Nanosheets while preserving their ∼ 1 nm thickness and (111) facets. This Pt‐enriched surface suppresses Cu dissolution, providing a robust design principle for high‐performance and durable electrocatalysts.

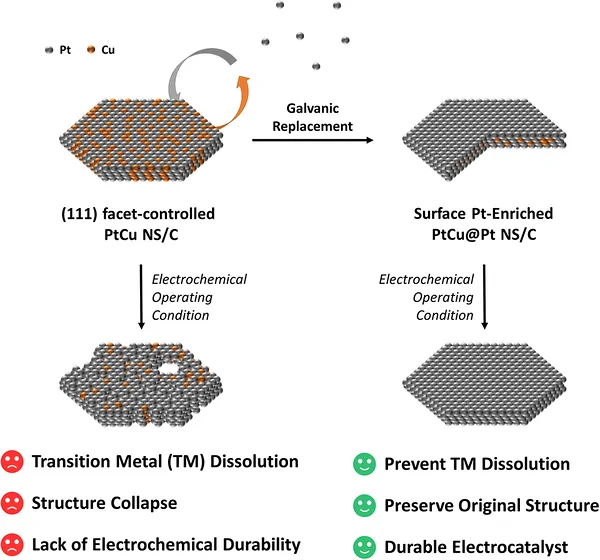

## 1 Introduction

Proton exchange membrane fuel cells (PEMFCs) have emerged as a promising energy conversion technology for sustainable transportation and portable power systems due to their high efficiency and zero‐emission operation [1, 2, 3, 4]. However, their widespread commercialization remains hindered by the sluggish kinetics of the oxygen reduction reaction (ORR) at the cathode and the high cost associated with Pt‐based catalysts. To address these challenges, extensive efforts have been devoted to improving both the activity and durability of Pt‐based electrocatalysts [5, 6, 7, 8].

Among various catalyst design strategies, alloying Pt with transition metals such as Cu, Co, and Ni has proven highly effective in enhancing ORR activity by modulating the electronic structure and inducing lattice strain effects [9, 10, 11, 12, 13]. Beyond compositional tuning, shape‐controlled Pt alloys exposing specific crystallographic facets have been actively explored to further boost ORR activity [14, 15, 16]. In this regard, two‐dimensional (2D) ultrathin nanosheets have attracted particular attention due to their high surface‐to‐volume ratios, abundant exposure of active facets, and tunable electronic properties [17]. These features enable superior catalytic performance compared to conventional nanoparticle‐based catalysts.

Despite these advantages, ultrathin Pt‐based nanosheets suffer from severe structural instability under electrochemical operating conditions. Their high surface energy and reduced thickness make them particularly susceptible to transition metal dissolution and structural degradation [17, 18, 19]. This intrinsic instability presents a critical barrier to their practical application in PEMFC systems.

To mitigate such degradation, the formation of a Pt‐enriched surface layer has been widely explored in Pt‐based alloy catalysts, as it can effectively suppress the dissolution of less noble metals while maintaining favorable electronic interactions with the alloy core [20, 21, 22]. While such surface modification strategies have been successfully implemented in nanoparticle systems through approaches such as thermal annealing, dealloying, and surface deposition, their application to ultrathin 2D nanosheets remains largely unexplored.

This limitation arises from the intrinsic structural fragility of ultrathin nanosheets. In this work, we find that conventional approaches based on high‐temperature reconstruction or additional metal deposition lead to severe structural degradation of nanosheets, including collapse into spherical particles or significant thickening, thereby compromising their anisotropic morphology and intrinsic advantages. Consequently, achieving surface‐selective Pt enrichment while preserving the ultrathin 2D architecture remains a critical challenge. Addressing this challenge would extend the concept of surface‐selective Pt enrichment engineering from conventional nanoparticles to ultrathin 2D alloy nanostructures, providing a general strategy for developing durable electrocatalysts for practical PEMFC applications.

Herein, we report a surface‐selective Pt enrichment strategy via galvanic replacement to stabilize ultrathin PtCu nanosheets while preserving their morphology and alloy characteristics. Unlike conventional approaches, galvanic replacement facilitates the preferential substitution of near‐surface Cu with Pt under mild conditions, thereby mitigating structural reconstruction or overgrowth. The resulting PtCu@Pt nanosheets retain their ultrathin 2D architecture (∼ 1 nm thickness) with a Pt‐enriched outer region and an alloyed PtCu subsurface, effectively suppressing Cu dissolution while maintaining beneficial electronic interactions. The PtCu@Pt nanosheets exhibit a favorable balance between well‐preserved activity and enhanced durability. The catalyst delivers high ORR activity with a mass activity of 1.71 A mg_Pt_^−1^ at 0.9 V_RHE_, while showing improved stability in both half‐cell and single‐cell PEMFC tests. Notably, the catalyst demonstrates minimal performance degradation after 30 000 accelerated stress cycles and outstanding voltage stability under practical PEMFC operating conditions. This work provides a viable strategy for stabilizing ultrathin 2D alloy nanostructures and offers a general design principle for developing durable, high‐performance electrocatalysts for PEMFC applications.

## 2 Results and Discussion

### 2.1 Synthesis and Characterization of PtCu@Pt NS/C

PtCu nanosheets supported on carbon (PtCu NS/C) were synthesized using carbon monoxide and iodide ions as structure‐directing agents [17, 23, 24], yielding uniformly dispersed ultrathin nanosheets with lateral dimensions of 10–15 nm and thicknesses below 1 nm (Figure S1A,B). Transmission electron microscopy (TEM) analysis confirms the formation of well‐defined two‐dimensional (2D) nanosheets, while selected‐area electron diffraction (SAED) and fast Fourier transform (FFT) patterns reveal a face‐centered cubic (FCC) structure with a preferential [111] orientation (Figure S1C,D), indicating anisotropic growth along the (111) plane.

The alloyed nature of PtCu NS/C is verified by X‐ray diffraction (XRD), where the (111) peak shifts from 39.5° for Pt/C (Figure S2) to 41.4° for PtCu NS/C (Figure 1B), corresponding to lattice contraction induced by Pt‐Cu alloying. Consistent with this observation, compositional analyses using energy‐dispersive X‐ray spectroscopy (EDS) and inductively coupled plasma optical emission spectroscopy (ICP‐OES) reveal atomic Pt:Cu ratios of 41:59 and 45:55, respectively (Figures S3D, S4C,D), confirming the formation of a Cu‐rich alloy phase.

**FIGURE 1:**
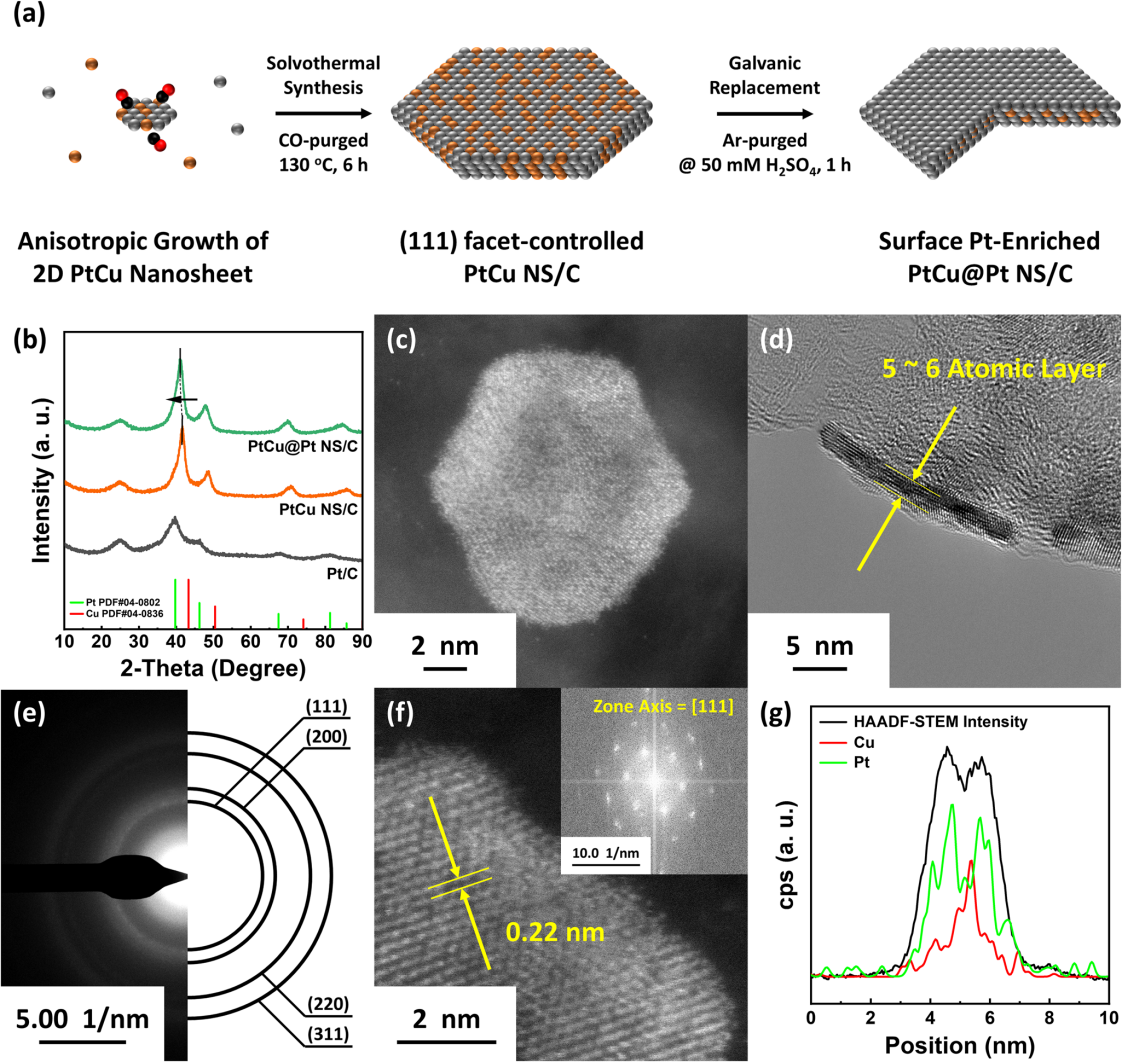
Structural characterization of PtCu@Pt NS/C. (a) Schematic illustration of the synthesis process. (b) XRD patterns of PtCu@Pt NS/C, PtCu NS/C, and Pt/C. (c) High‐magnification top‐view HAADF‐STEM image, (d) side‐view bright‐field TEM image, (e) SAED pattern ring, (f) enlarged top‐view HAADF‐STEM image and corresponding FFT image, and (g) EDS line scanning profiles of PtCu@Pt NS/C.

To stabilize the nanosheet surface against electrochemical degradation, galvanic replacement was employed to preferentially replace near‐surface Cu atoms with Pt (Figure 1A). Notably, the resulting PtCu@Pt NS/C retains the ultrathin nanosheet morphology without observable thickening or aggregation, preserving both lateral dimensions (∼ 10–15 nm) and thickness (∼ 1 nm) (Figure 1C,D). The FCC structure and [111] orientation are also maintained, as confirmed by SAED and FFT analyses (Figure 1E,F), indicating that the replacement process proceeds without inducing structural reconstruction.

The XRD pattern of PtCu@Pt NS/C exhibits diffraction peaks located between those of Pt/C and PtCu NS/C (Figure 1B), suggesting partial relaxation of lattice contraction due to Pt enrichment. Correspondingly, the Pt content increases significantly to 70:30 (EDS) and 68:32 (ICP‐OES) (Figures S3B, S4A,B). The Pt:Cu ratio measured by ICP‐OES closely matches the theoretical value predicted for a five‐atomic‐layer nanosheet assuming surface‐selective atom exchange, based on the nanosheet thickness determined by TEM. Importantly, this compositional evolution occurs without any measurable increase in nanosheet thickness, indicating that Pt incorporation preferentially occurs in near‐surface regions rather than resulting from bulk alloy restructuring or additional Pt overgrowth.

This thickness invariance provides key mechanistic insight into the galvanic replacement process. Unlike conventional Pt deposition or thermal annealing, which typically induces particle growth or structural collapse, galvanic replacement can facilitate preferential near‐surface Pt incorporation under mild conditions. Under the present reaction conditions, Pt incorporation reaches a saturation level with increasing Pt‐precursor addition while the ultrathin 2D architecture is preserved.

To further elucidate why this approach is uniquely effective for ultrathin nanosheets, we compared it with representative strategies for generating Pt‐enriched surfaces (Figure S5). Secondary Pt deposition [25] results in a mixture of nanospheres and thickened nanosheets (Figure S5A), which compromises the structural integrity and surface area of the nanosheets. Similarly, post‐synthetic treatments commonly applied to nanoparticles [21, 26], such as acid leaching followed by high‐temperature annealing (> 400°C), lead to the transformation of ultrathin 2D nanosheets into spherical particles (Figure S5B), reflecting the thermodynamic instability of such ultrathin structures under high‐temperature conditions. In addition, attempts to achieve Pt‐enriched surfaces by increasing the Pt precursor concentration during synthesis result in uncontrolled particle growth, yielding a heterogeneous mixture of nanospheres, nanoflakes, thick nanosheets, and irregular polyhedral structures (Figure S5C).

To further investigate the controllability of the galvanic replacement process, a series of control samples was prepared by varying the stoichiometric amount of Pt precursor relative to the theoretical number of surface Cu atoms (Table S1). As the target exchange ratio increased from 25% to 50% and 100%, the Pt:Cu atomic ratio and Pt content increased correspondingly, accompanied by a systematic negative shift of the PtCu (111) diffraction peak (Figure S6), indicating the progressive incorporation of Pt into the nanosheet framework.

Importantly, despite these compositional changes, TEM analysis confirmed that all samples retained their ultrathin 2D morphology without detectable structural deformation or pore formation (Figure 1C,D, Figures S1A,B and S7). When an excess amount of Pt precursor corresponding to a 200% target exchange ratio was introduced, no further increase in the Pt:Cu ratio or Pt content was observed. This saturation behavior is consistent with preferential near‐surface Pt incorporation rather than continuous Pt deposition.

The time‐resolved elemental profiles reveal that, in the presence of the Pt precursor, the Pt uptake occurs simultaneously with the Cu release immediately following the removal of surface copper oxides, consistent with a coupled redox‐driven replacement process (Figure S8). Conversely, the control system without the Pt precursor undergoes extensive and non‐selective Cu dissolution across the entire nanosheet framework rather than a localized surface passivation. This distinct kinetic behavior, together with the compositional and structural analyses, supports galvanic replacement as a plausible pathway for preferential near‐surface Pt enrichment while preserving the underlying 2D morphology.

Under ambient acidic conditions, the transformation from PtCu NS/C to PtCu@Pt NS/C could potentially involve galvanic replacement, Cu dissolution, internal reconstruction, or dissolution‐redeposition. Internal reconstruction alone cannot account for the observed compositional change because atomic redistribution does not alter the overall Pt:Cu ratio or Pt content. Although selective Cu dissolution can increase the relative Pt content, the Pt wt.% expected from Cu dissolution alone (17.1 wt.%) is lower than the experimentally measured value of 22.0 wt.% (Table S1), supporting the incorporation of Pt from the external precursor. While localized dissolution–redeposition cannot be completely excluded by the present *ex‐situ* analyses, the combined observations of external Pt incorporation, preferential Pt enrichment near the nanosheet surfaces, and preservation of a smooth, pinhole‐free 2D morphology support surface‐selective galvanic replacement as a plausible predominant pathway.

These observations collectively highlight a fundamental limitation of conventional approaches to surface Pt enrichment: they rely on high‐temperature reconstruction or additional metal deposition, both of which are inherently incompatible with preserving ultrathin 2D morphologies. Under the present reaction conditions, preferential near‐surface Pt enrichment occurs at low temperature while effectively preserving the structural integrity of the nanosheets.

### 2.2 Structural and Electronic Configuration of the Pt‐Enriched Surface

The surface structure of PtCu@Pt NS/C was systematically investigated using complementary spatially resolved and spectroscopic analyses to elucidate the nature of Pt enrichment induced by galvanic replacement. High‐resolution cross‐sectional side‐view HAADF‐STEM imaging, together with corresponding intensity profiles and EDS line‐scan analysis, was employed to directly examine the elemental distribution across the nanosheet thickness. According to the Z‐contrast principle, in which the HAADF‐STEM intensity is proportional to the square of the atomic number [27], pristine PtCu NS/C exhibits a single intensity maximum across the nanosheet thickness, consistent with a homogeneous alloy distribution (Figures S9 and S10). In contrast, PtCu@Pt NS/C generally exhibits broadened intensity distributions, with dual maxima observed in several profiles near the outer regions. These features are consistent with the Pt distributions observed in the corresponding HAADF‐STEM intensity profiles (Figures S11, S12 and Figure 1G). While these combined *ex‐situ* profiles do not directly confirm a continuous, conformal Pt layer across both surfaces, they support preferential near‐surface Pt enrichment relative to the homogeneous alloy core.

Additional structural evidence is provided by strain analysis derived from high‐resolution transmission electron microscopy (HRTEM) images [28]. The in‐plane strain distribution (ε_xy_), obtained using the Strain++ software [29, 30, 31], was reproducibly analyzed for three independent nanosheets of each catalyst. PtCu@Pt NS/C consistently exhibits a uniformly distributed compressive strain (Figure S13), whereas PtCu NS/C shows only weak, noise‐like strain features (Figure S14). Such compressive strain can be attributed to lattice mismatch between the Pt‐enriched outer region and the underlying PtCu alloy [32, 33], further supporting the formation of a surface‐modified structure.

Surface‐sensitive X‐ray photoelectron spectroscopy (XPS) analysis further confirms the compositional redistribution. The surface Pt:Cu ratio increases from 58:42 in PtCu NS/C to 77:23 in PtCu@Pt NS/C (Figure S15 and Table S2), indicating substantial Pt enrichment at the surface. Correspondingly, the Pt 4f_7/2_ binding energy shifts slightly from 71.6 eV for PtCu NS/C to 71.7 eV for PtCu@Pt NS/C (Figure S16A). This positive shift is consistent with the reduced alloying effect at the outermost surface as the local coordination environment becomes more Pt‐rich following galvanic replacement. Nevertheless, the Pt 4f binding energy of PtCu@Pt NS/C remains lower than that of commercial Pt/C (71.9 eV), indicating that the underlying PtCu subsurface continues to influence the electronic structure of the surface Pt layer through ligand and lattice strain effects. The retained electronic interaction between the Pt‐enriched surface and the PtCu subsurface is expected to contribute to the high ORR activity while improving structural stability.

Consistent with this interpretation, Cu 2p spectra show a significant reduction in Cu(II) species and the disappearance of characteristic satellite peaks in PtCu@Pt NS/C (Figure S16B), indicating effective suppression of surface Cu oxidation. Meanwhile, the unchanged Cu(0) binding energy confirms that Cu remains in a metallic state within the subsurface region, rather than being completely removed.

X‐ray absorption near‐edge structure (XANES) analysis further supports the preservation of alloy electronic characteristics. Both PtCu NS/C and PtCu@Pt NS/C exhibit lower white‐line intensities at the Pt L_3_‐edge compared to Pt foil (Figure S17), indicating increased Pt 5d electron density due to electron donation from Cu [34, 35, 36]. In addition, Cu K‐edge XANES analysis (Figure 2A and Figure S18) reveals an increased fraction of metallic Cu in PtCu@Pt NS/C, confirming that Cu atoms are predominantly retained in a reduced, subsurface coordination environment.

**FIGURE 2:**
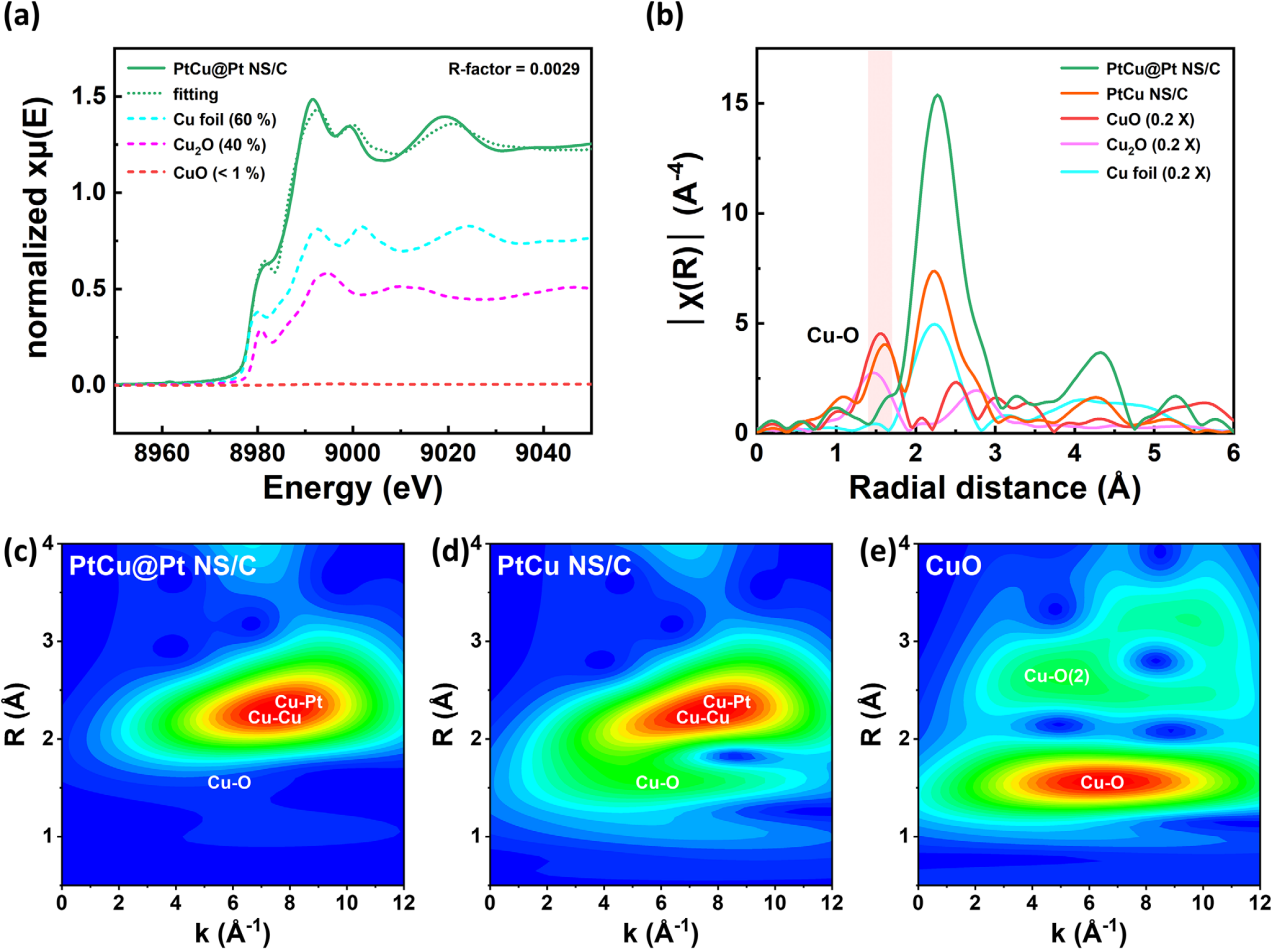
Structural and electronic configuration of the Pt‐enriched surface in PtCu@Pt NS/C. (a) Cu K‐edge XANES spectra of PtCu@Pt NS/C. The dotted line represents linear combination fitting of Cu species. (b) k^3^‐weighted Cu K‐edge EXAFS spectra of PtCu@Pt NS, PtCu NS/C, CuO, Cu_2_O, and Cu foil. k^3^‐weighted wavelet transformed Cu K‐edge EXAFS spectra of (c) PtCu@Pt NS/C, (d) PtCu NS/C, and (e) CuO.

Extended ‐ray absorption fine structure (EXAFS) analysis provides quantitative insight into local coordination environments. Compared to PtCu NS/C, PtCu@Pt NS/C exhibits reduced Pt‐Cu coordination and enhanced Pt‐Pt contributions (Figures S19 and S20), indicating partial surface enrichment of Pt. Simultaneously, the Pt‐Pt bond, which is negligible in PtCu NS/C, slightly appears in PtCu@Pt NS/C at around 2.7 Å, supporting the formation of Pt‐rich surface layer (Table S3). Importantly, Cu‐O coordination features observed in PtCu NS/C are absent in PtCu@Pt NS/C (Figure 2B and Scheme S1), indicating that surface‐exposed Cu is effectively suppressed.

Wavelet transform EXAFS (WT‐EXAFS) analysis further distinguishes these structural features (Figure S21). While both samples retain Pt‐Cu scattering signatures indicative of alloying, PtCu NS/C exhibits distinct Cu‐O contributions in the 1–2 Å region, whereas this feature is absent in PtCu@Pt NS/C (Figure 2C–E). Instead, PtCu@Pt NS/C shows broadened metallic scattering features consistent with Cu atoms embedded in a Pt‐coordinated environment (Figure S22) beneath a Pt‐enriched surface.

Taken together, these multi‐scale analyses consistently support a structural model in which PtCu@Pt NS/C comprises a Pt‐enriched outer region coupled with an alloyed PtCu subsurface. Although the Pt‐enriched region is ultrathin and cannot be directly resolved as a discrete shell, the combined evidence, including thickness invariance, surface‐sensitive compositional redistribution, suppression of Cu oxidation, and preserved alloy electronic structure, supports preferential near‐surface Pt enrichment that effectively shields subsurface Cu while maintaining the ultrathin nanosheet architecture.

### 2.3 Catalytic ORR Activity and Durability of PtCu@Pt NS/C

The ORR performance of PtCu@Pt NS/C, PtCu NS/C, and commercial Pt/C was first evaluated in an O_2_‐saturated 0.1 m HClO_4_ electrolyte using a rotating disk electrode (Figure 3A). PtCu@Pt NS/C exhibits an onset potential of 1.018 V_RHE_, comparable to PtCu NS/C (1.020 V_RHE_) and significantly higher than Pt/C (0.997 V_RHE_). Similarly, the half‐wave potential (E_1/2_) of PtCu@Pt NS/C reaches 0.928 V_RHE_, slightly lower than PtCu NS/C (0.933 V_RHE_) but markedly higher than Pt/C (0.884 V_RHE_), indicating that the high ORR activity of PtCu nanosheets is largely retained after surface modification.

**FIGURE 3:**
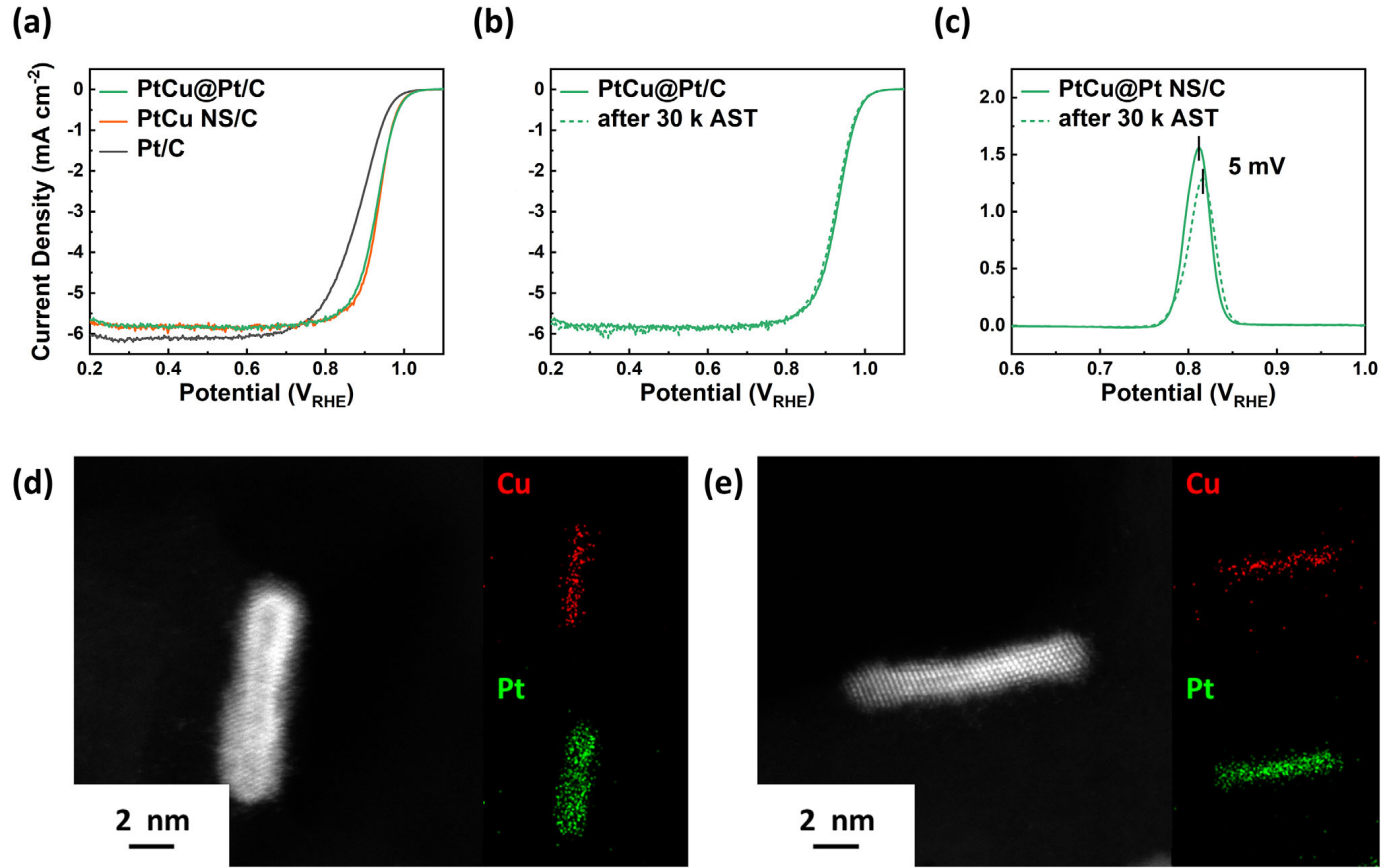
Half‐cell ORR activity and durability of PtCu@Pt NS/C. (a) ORR polarization curves of PtCu@Pt NS/C, PtCu NS/C, and Pt/C. (b) LSV curves and (c) CO stripping peak shift of PtCu@Pt NS/C after 30 000 AST cycles. Side view HAADF‐STEM image and corresponding EDS mapping image of (d) PtCu@Pt NS/C and (e) PtCu NS/C after 30 000 AST cycles.

In terms of mass activity, PtCu@Pt NS/C delivers 1.71 A mg_Pt_^−1^ at 0.9 V_RHE_, which is moderately lower than that of PtCu NS/C (2.23 A mg_Pt_^−1^) but more than 4.2 times higher than commercial Pt/C (0.41 A mg_Pt_^−1^). To decouple intrinsic activity from surface area effects, the electrochemically active surface area (ECSA) was determined using CO stripping instead of cyclic voltammetry (Figures S23 and S24) [37, 38]. The ECSA values are comparable across all catalysts (70, 74, and 78 m^2^ g_Pt_^−1^ for PtCu@Pt NS/C, PtCu NS/C, and Pt/C, respectively), indicating that the observed activity trends originate primarily from differences in intrinsic catalytic properties.

Accordingly, the specific activity of PtCu@Pt NS/C (2.44 mA cm^−2^) remains higher than that of Pt/C (0.53 mA cm^−2^), while being lower than PtCu NS/C (3.01 mA cm^−2^). This concomitant decrease can be attributed to partial attenuation of the ligand and strain effects arising from reduced surface Cu exposure following surface Pt enrichment. Nevertheless, the preserved electronic interaction with subsurface Cu, as evidenced by spectroscopic analyses, enables PtCu@Pt NS/C to maintain substantially enhanced intrinsic activity compared to pure Pt catalysts.

Kinetic analysis further supports this trend. PtCu@Pt NS/C exhibits a Tafel slope of 52 mV dec^−1^, slightly higher than PtCu NS/C (48 mV dec^−1^) but significantly lower than Pt/C (63 mV dec^−1^) (Figure S25), indicating favorable reaction kinetics. In addition, rotating ring‐disk electrode (RRDE) measurements confirm that all catalysts follow a dominant four‐electron ORR pathway with negligible H_2_O_2_ formation (Figures S26 and S27), indicating that surface modification does not compromise reaction selectivity.

While PtCu@Pt NS/C shows only a marginal decrease in activity relative to PtCu NS/C, its primary advantage lies in enhanced durability under electrochemical stress. To evaluate long‐term stability, accelerated stress tests (ASTs) were conducted using square‐wave potential cycling between 0.60 and 0.95 V_RHE_. After 30 000 AST cycles, PtCu@Pt NS/C exhibits minimal degradation, with negligible onset potential shift and only a 3 mV loss in E_1/2_, compared to larger shifts observed for PtCu NS/C (7 mV) and Pt/C (25 mV) (Figures S28 and S29). In terms of mass activity, PtCu@Pt NS/C retains 82.5% of its initial value (1.41 A mg_Pt_^−1^), whereas PtCu NS/C and Pt/C exhibit significantly greater losses of 29.1% and 34.1%, respectively (Figure 3B, Figures S28A, S29A, and S30A).

Consistent with these results, PtCu@Pt NS/C shows improved surface stability, with only a 12.9% loss in ECSA after AST, compared to ∼ 24% losses for PtCu NS/C and Pt/C (Figure 3C, Figures S28B, S29B, and S30B). Furthermore, CO stripping measurements reveal only a minor peak shift (5 mV) for PtCu@Pt NS/C, whereas PtCu NS/C and Pt/C exhibit substantial shifts of 32 and 23 mV, respectively, indicating significant surface reconstruction in the latter cases [38, 39]. Accordingly, PtCu@Pt NS/C exhibits a minimal loss in specific activity of only 5.4% (2.31 mA cm^−2^), which is lower than that of PtCu NS/C (6.4%, 2.82 mA cm^−2^) and significantly smaller than that of Pt/C (12.9%, 0.46 mA cm^−2^) (Figure S30C).

Post‐AST structural analysis provides direct insight into the origin of this enhanced durability. While Pt/C shows pronounced particle aggregation (Figure S31), both nanosheet catalysts largely retain their morphology (Figure S32). However, PtCu NS/C exhibits noticeable perforations and a substantial compositional shift (Pt:Cu from 45:55 to 70:30) (Figures S3D, S4C,D, and S33C,D), indicating severe Cu dissolution. In contrast, PtCu@Pt NS/C shows minimal structural degradation and only a slight compositional change (70:30 to 75:25), demonstrating effective suppression of Cu leaching (Figures S3B, S4A,B, and S33A,B).

This difference is further supported by electron microscopy and elemental mapping. PtCu@Pt NS/C maintains a distinct compositional contrast between the Pt‐enriched outer region and the PtCu interior after AST (Figure 3D), whereas PtCu NS/C shows no such differentiation (Figure 3E). Furthermore, post‐AST cross‐sectional HAADF‐STEM imaging and corresponding intensity profiles show that the preferential near‐surface Pt‐enrichment is retained after durability testing (Figure S34). In addition, ICP‐MS analysis confirms significantly reduced Cu dissolution into the electrolyte for PtCu@Pt NS/C compared to PtCu NS/C (Figure S35).

To further clarify the dominant degradation pathway, post‐mortem XRD and XPS analyses were performed on catalysts spray‐coated onto carbon paper before and after 10 000 AST cycles. The XRD patterns of PtCu NS/C exhibit a noticeable negative shift of the main diffraction peak after AST, indicating a reduced alloying effect caused by transition‐metal dissolution. In contrast, PtCu@Pt NS/C shows little change in the diffraction profile, suggesting that the Pt‐enriched surface effectively suppresses alloy degradation (Figure S36).

This interpretation is further supported by post‐mortem Pt 4f XPS analysis (Figures S37 and S38). The Pt 4f peak of PtCu NS/C shifts from 71.6 to 71.7 eV after AST, consistent with the reduced alloying effect resulting from Cu dissolution. In contrast, the Pt 4f binding energy of PtCu@Pt NS/C remains essentially unchanged at 71.7 eV, while commercial Pt/C also shows negligible variation at 71.9 eV (Figures S39 and S40). Furthermore, the surface Pt:Cu ratio of PtCu NS/C changes from 58:42 to 75:25 after AST, whereas PtCu@Pt NS/C exhibits only a negligible change from 77:23 to 76:24 (Tables S4 and S5). These results demonstrate that the Pt‐enriched surface effectively suppresses Cu dissolution and preserves the alloy composition during electrochemical operation.

Collectively, these results indicate that surface Pt enrichment effectively stabilizes the nanosheet structure by suppressing Cu dissolution and mitigating surface reconstruction. Compared with previously reported Pt‐alloy catalysts, PtCu@Pt NS/C exhibits substantial compositional retention with only a minimal decrease in transition‐metal atomic fraction after durability testing (Table S6). Direct quantitative comparison should, however, be made with caution because catalyst compositions and AST protocols differ among studies. As a result, PtCu@Pt NS/C achieves a favorable balance between high intrinsic activity and substantially enhanced durability, highlighting the effectiveness of galvanic replacement in stabilizing ultrathin Pt‐based nanosheet catalysts under realistic electrochemical conditions.

To evaluate the practical applicability of the catalysts in PEMFCs, membrane‐electrode assemblies (MEAs) were fabricated using PtCu@Pt NS/C, PtCu NS/C, and commercial Pt/C as cathode catalysts with a Pt loading of 0.05 mg_Pt_ cm^−2^. Commercial Pt/C (60 wt.%) was used as the anode catalyst at a loading of 0.10 mg_Pt_ cm^−2^, and a Nafion 212 membrane served as the electrolyte. The MEAs were assembled with gas diffusion layers and graphite bipolar plates and operated at 65°C under fully humidified H_2_ and O_2_.

Figure 4A shows the initial single‐cell polarization curves. Both PtCu@Pt NS/C and PtCu NS/C exhibit superior performance compared to commercial Pt/C across the entire current density range. At a low current density of 0.2 A cm^−2^, where performance is predominantly governed by cathode kinetics, PtCu@Pt NS/C and PtCu NS/C deliver cell voltages of 730 and 734 mV, respectively, significantly higher than that of Pt/C (688 mV). These results indicate that surface modification via galvanic replacement largely preserves the high intrinsic activity of PtCu nanosheets under practical operating conditions.

**FIGURE 4:**
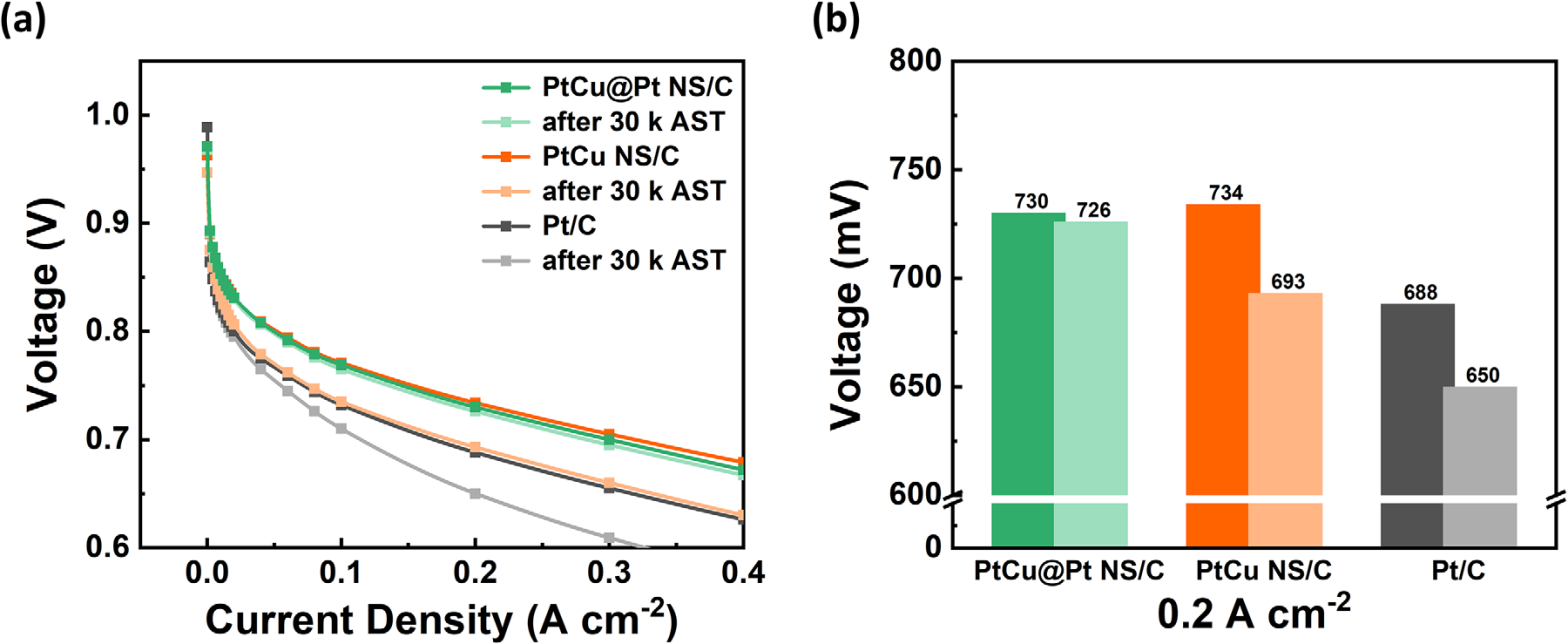
Single‐cell performance and durability of PtCu@Pt NS/C. (a) Current‐voltage polarization curves of PtCu@Pt NS/C, PtCu NS/C, and Pt/C before and after 30 000 AST cycles. (b) Voltage loss at 0.2 A cm^−2^ after 30 000 AST cycles.

To assess durability under realistic stress conditions, accelerated stress tests (ASTs) were conducted using square‐wave potential cycling between 0.60 and 0.95 V, with a 3 s hold at each potential [40]. After 30 000 AST cycles (Figure 4B and Figure S41), PtCu@Pt NS/C exhibits outstanding voltage stability, with only a 4 mV loss at 0.2 A cm^−2^, whereas PtCu NS/C and Pt/C show significantly larger degradations of 41 and 38 mV, respectively. The durability advantage becomes more evident at a high current density of 1.2 A cm^−2^, where the cell voltage of PtCu@Pt NS/C decreases only slightly from 480 to 457 mV after 30 000 AST cycles. In contrast, PtCu NS/C and commercial Pt/C exhibit substantially larger voltage losses, decreasing from 508 to 386 mV and from 450 to 360 mV, respectively (Figure S42). Furthermore, under the more practical H_2_‐Air operating conditions, PtCu@Pt NS/C retains its polarization performance after 30 000 AST cycles, whereas both PtCu NS/C and commercial Pt/C show pronounced performance degradation over the entire current‐density range (Figure S43). This substantial difference highlights the superior durability of PtCu@Pt NS/C at the single‐cell level. Moreover, PtCu@Pt NS/C demonstrates durable performance under practical single‐cell PEMFC conditions. Compared with previously reported PGM‐based 2D nanosheet catalysts, which are largely limited to half‐cell evaluation, our work provides comprehensive validation from half‐cell measurements to device‐level durability testing (Table S7).

To elucidate the origin of this enhanced durability, post‐mortem analyses were performed on the cathode catalysts after AST cycling. STEM–EDS mapping reveals that the Pt:Cu atomic ratio of PtCu@Pt NS/C remains nearly unchanged (70:30 → 72:28), whereas PtCu NS/C undergoes severe compositional degradation (41:59 → 84:16), indicating extensive Cu leaching during operation (Figure S44). X‐ray fluorescence (XRF) analysis further corroborates these results, showing minimal compositional change for PtCu@Pt NS/C (74:26) but pronounced Cu depletion for PtCu NS/C (82:18).

Further evidence of Cu migration was obtained by cross‐sectional analysis of the MEAs. For PtCu@Pt NS/C, no detectable Cu signal is observed within the membrane before or after AST cycling. In contrast, PtCu NS/C exhibits clear Cu signals inside the membrane after AST (Figure S45), indicating significant Cu dissolution and subsequent migration into the electrolyte membrane.

Electrochemical impedance spectroscopy (EIS) analysis further reveals that this uninhibited Cu leaching is associated with impaired proton transport within the catalyst layer, consistent with ionomer contamination by dissolved Cu species [41, 42]. Accordingly, the catalyst layer proton transport resistance (R_ion_) of PtCu NS/C increased by 38.5% after 30 000 AST cycles, whereas PtCu@Pt NS/C shows only a 10% change (Figure S46 and Table S8).

These findings collectively indicate that surface Pt enrichment effectively suppresses Cu leaching and mitigates catalyst degradation under operating conditions. At the same time, the preservation of the nanosheet morphology and preferential (111) facet enables the retention of high catalytic activity. As a result, PtCu@Pt NS/C achieves a favorable balance between performance and durability, demonstrating strong potential as a practical cathode catalyst for PEMFC applications.

## 3 Conclusion

In this work, we present a strategy to stabilize ultrathin Pt‐based nanosheet catalysts through surface‐selective Pt enrichment via galvanic replacement. The resulting PtCu@Pt NS/C retains the characteristic ultrathin two‐dimensional morphology with a thickness of ∼ 1 nm and dominant (111) facet exposure, while exhibiting a Pt‐enriched outer region coupled with an alloyed PtCu subsurface. Comprehensive structural and spectroscopic analyses reveal that the surface‐selective substitution of Cu with Pt effectively suppresses surface Cu exposure without disrupting the underlying alloy structure or nanosheet integrity. As a result, PtCu@Pt NS/C maintains high intrinsic ORR activity, with only a marginal decrease compared to PtCu NS/C, while significantly outperforming commercial Pt/C. More importantly, the Pt‐enriched surface markedly enhances electrochemical durability. PtCu@Pt NS/C exhibits substantially improved stability in both half‐cell and single‐cell PEMFC tests, showing minimal performance degradation after 30 000 accelerated stress test cycles. Post‐mortem analyses indicate that this enhanced durability is associated with the effective suppression of Cu dissolution and mitigation of surface reconstruction, which are critical degradation pathways in Pt‐based alloy catalysts.

Beyond the specific system studied here, this work highlights a fundamental limitation of conventional Pt‐enrichment strategies for ultrathin 2D nanostructures and demonstrates that galvanic replacement provides a viable route to achieve surface stabilization without compromising morphology or active site characteristics. The insights gained from this study offer a general design principle for developing durable, high‐performance electrocatalysts based on ultrathin nanosheet architectures for practical PEMFC applications.

## Author Contributions

**HyunWoo J. Yang**: conceptualization, methodology, formal analysis, data curation, visualization, investigation, Writing – original draft, validation. **SangJae Lee**: conceptualization, methodology, formal analysis, visualization, investigation, validation. **KwangHo Lee**: methodology, investigation, formal analysis, visualization, data curation. **HyunWoo Chang**: methodology, investigation, visualization, formal analysis. **HyunWoo T. Yang**: investigation, methodology, visualization, formal analysis. **Dongwon Shin**: formal analysis. **MinJun Kim**: formal analysis. **EunAe Cho**: writing – review and editing, writing – original draft, resources, supervision, project administration, software, funding acquisition.

## Funding

This work was supported by the Korea Institute of Energy Technology Evaluation and Planning (KETEP) funded by the Ministry of Climate, Energy and Environment (RS‐2021‐KP002516), and the National Research Foundation of Korea (NRF) funded by the Ministry of Science and ICT (RS‐2024‐00466554).

## Conflicts of Interest

The authors declare no conflicts of interest.

## Supporting information




**Supporting File**: advs78520‐sup‐0001‐SuppMat.docx.

## Data Availability

The data that support the findings of this study are available from the corresponding author upon reasonable request.
