# Supplementary material for: Surface‐Selective Pt Enrichment for Stabilizing Ultrathin 2D PtCu Nanosheets Toward Durable Oxygen Reduction
Source: Adv Sci (Weinh). 2026 Oct 27:e78520. Online ahead of print. doi: 10.1002/advs.78520 (PMC13712395; doi:10.1002/advs.78520)
Supplement: Supplementary file 1 [file ADVS-9999-e78520-s001.docx]

Supporting Information

Surface-Selective Pt Enrichment for Stabilizing Ultrathin 2D PtCu Nanosheets toward Durable Oxygen Reduction

HyunWoo J. Yang, SangJae Lee, KwangHo Lee, HyunWoo Chang, HyunWoo Yang, Dongwon Shin, MinJun Kim and EunAe Cho^*^

Experimental Methods

*Materials*

All chemicals were used without additional purification. Platinum(II) acetylacetonate (Pt(acac)_2_), copper(II) acetylacetonate (Cu(acac)_2_), potassium iodide (KI), poly vinyl pyrrolidone (PVP, molecular weight = 40,000), potassium tetrachloroplatinate (K_2_PtCl_4_), ascorbic acid, formamide, sulfuric acid (H_2_SO_4_), Nafion® solution (5 wt.%) were purchased from Sigma Aldrich. Commercial Pt/C (20 wt.%, TEC10V20E) were purchased from TANAKA Precious Metals. Ethanol (99.5%), acetone (99.5%), 1-propanol (NPA), 2-propanol (IPA), perchloric acid (HClO_4_) were purchased from Samchun Chemicals. Deionized water (DI, 18.2 MΩ cm) was purified through Millipore Direct-Q 5 UV water purification system.

*Catalyst Preparation*

The synthesis process was carried out with slight modifications to the methods used in previous studies. 80 mg of Pt(acac)_2_, 53.2 mg of Cu(acac)_2_, 600 mg of KI, 600 mg of ascorbic acid, 50 mg of PVP (molecular weight = 40,000) is dissolved in 40 mL of formamide vigorously to prepare the metal precursor solution. The prepared solution was transferred into a 50 mL vial with a pressure cap, purged with carbon monoxide (CO) gas for 30 minutes and then kept at 110 ℃ for 1 hour , then the temperature was raised to 130 ℃ and held for 6 hours and cooled down to room temperature. PtCu NS precipitates were collected with a centrifuge at 10,000 rpm and washed with a mixture of DI and acetone. Then PtCu nanosheets black were dispersed in 100 mL of DI, whereas 150 mg of Ketjen Black was dispersed in a mixture of 300 mL of DI and 100 mL of ethanol. PtCu nanosheets dispersion was added dropwise into the Ketjen black dispersion under vigorous stirring at 1,000 rpm. 200 µL of 0.5 M H_2_SO_4_ solution was added to adjust pH. Finally, the resulting product was filtered and washed with DI to obtain PtCu NS/C. After drying in vacuum oven, 200 mg of PtCu NS/C could be obtained.

50 mg of PtCu NS/C was dispersed in 50 mL of DI and sonication was performed for 30 minutes. PtCu NS/C dispersion was added to 400 mL Ar-sat. 0.05 M H_2_SO_4_ solution. 20.8 mg of K_2_PtCl_4_ was dispersed in 50 mL of DI and added into prepared solution dropwise with vigorous stirring of 1,000 rpm. Solution was kept for 1 hour and the product was collected with vacuum filtering and washed with DI. After Drying in vacuum oven, PtCu@Pt NS/C could be collected.

*Material Characterizations*

X-ray diffraction (XRD) spectra of samples were obtained by high-resolution powder X-ray diffractometer (SmartLab) with Cu K-α spectrum (λ = 1.5406 Å). Transmission electron microscopic (TEM) images were acquired by 300 kV Field Emission TEM (Tecnai G2 F30 S-Twin). High-resolution transmission electron microscopic (HRTEM) and high-angle annular dark-field scanning transmission electron microscopic (HAADF-STEM) images were obtained by a double Cs-corrected TEM (Titan cubed G2 60-300, 300 kV). To analyze atomic distribution on particles, energy dispersive spectroscopy (EDS) mapping images were acquired for 180 seconds with 200 kV Field Emission TEM (Talos F200X). For the high-magnification STEM-EDS mapping analysis, at least five independent particles were analyzed, and the average composition was used for quantitative analysis. Metal composition and its atomic ratio were measured with inductively coupled plasma optical emission spectroscopy (ICP-OES, Agilent 5110). To confirm that effective replacement was occurring during the galvanic replacement reaction and to clarify the underlying kinetic mechanism, 2 mL samples were collected from the electrolyte at 3-minute intervals, diluted in aqua regia, and systematically analyzed using ICP-OES. Surface electronic states of the samples were identified using X-ray photoelectron spectroscopy (XPS, Sigma Probe). The binding energies were calibrated by referencing the adventitious C 1s peak to 284.8 eV to correct for surface charging. High-resolution Pt 4f and Cu 2p spectra were fitted using Thermo Scientific Avantage software after applying a Smart background subtraction. The Pt:Cu atomic ratio was quantified using the built-in Scofield relative sensitivity factors (RSFs) (16.678 for Pt 4f and 26.513 for Cu 2p spectra), which account for the instrument-specific transmission function and the TPP-2M inelastic mean free path model. X-ray fluorescence spectrometer (XRF, ZSX Primus II) was utilized for bulk composition analysis of the MEA. The cross-section of MEA was cut through cryo-milling (ArBlade5000), and the elemental composition of the corresponding cross-section was analyzed through field emission scanning electron microscope (JSM-IT800).

Geometric phase analysis (GPA) was performed using HAADF-STEM images acquired along the [111] zone axis to minimize lattice distortions caused by sample tilting. Fast Fourier transform (FFT) patterns exhibited six first-order {220} reflections of the face-centered cubic lattice, from which two non-collinear reflections separated by 60^o^ (or 120^o^) were selected as the g_1_ and g_2_ vectors. Digital masks were applied to isolate the corresponding Fourier components, and the reciprocal vectors were refined using the phase-gradient relationship (Δg = 1/2π ▽P_g_). The displacement fields (u_x_, u_y_) were then calculated by inverting the g-vector matrix, and the strain tensor (ε = ½(e+e^T^)) was obtained from the local distortion matrix. The resulting strain maps were visualized as RGB images using the Strain++ software.

X-ray absorption spectroscopy (XAS) experimental data have been gathered at the 8C-Nano XAFS beamline of the Pohang Light Source-II (PLS-II), Pohang Accelerator Laboratory. The monochromatic X-rays were generated with a Si (111) double-crystal monochromator and its intensity was adjusted to minimize the contribution from the harmonics. The beam was further shaped by a secondary source aperture which provides a spot size of 0.5 mm (vertical) x 1 mm (horizontal). The radiation was from a tapered in-vacuum undulator; thus the measurements can be made in both the transmission and fluorescence detection modes. The Athena program of the IFEFFIT software suite was used to process the spectra that were obtained. To obtain χ(k), the post-edge background subtraction and edge-step normalization were used. The χ(k) functions weighted by k³ were further Fourier transformed into R space with a Hanning window to separate the extended X-ray absorption fine structure (EXAFS) contributions from the different coordination shells. The coordination numbers and bond distances were finally gotten through the help of the Artemis module which is part of the IFEFFIT package by means of fitting the EXAFS curves to the standard EXAFS formula.[1-2]

$$\boldsymbol{\chi}\left( \mathbf{k} \right)\boldsymbol{=}\sum_{\boldsymbol{j}} \frac{\boldsymbol{N}_{\boldsymbol{j}}\boldsymbol{S}_{\boldsymbol{0}}^{\boldsymbol{2}}}{\boldsymbol{k}\boldsymbol{R}_{\boldsymbol{j}}^{\boldsymbol{2}}}\boldsymbol{F}_{\boldsymbol{j}}\boldsymbol{(k)}\boldsymbol{e}^{\boldsymbol{-}\frac{\boldsymbol{2}\boldsymbol{Rj}}{\boldsymbol{\lambda}_{\boldsymbol{j}}\left( \boldsymbol{k} \right)}}\boldsymbol{e}^{\boldsymbol{-2}\boldsymbol{k}^{\boldsymbol{2}}\boldsymbol{\sigma}_{\boldsymbol{j}}^{\boldsymbol{2}}}\mathbf{sin}\boldsymbol{[2}\boldsymbol{k}\boldsymbol{R}_{\boldsymbol{j}}\boldsymbol{+}\boldsymbol{\Phi}_{\boldsymbol{j}}\boldsymbol{(k)]}$$

$$\mathbf{k=}\frac{\boldsymbol{2}\boldsymbol{\pi}}{\boldsymbol{\lambda}}\boldsymbol{=}\sqrt{\frac{\boldsymbol{2}\boldsymbol{m}_{\boldsymbol{e}}\boldsymbol{(E-}\boldsymbol{E}_{\boldsymbol{0}}\boldsymbol{)}}{\boldsymbol{\hbar}^{\boldsymbol{2}}}}$$

*Half-cell Electrochemical Characterizations*

Electrochemical performance and its durability were measured in a three-electrode system using a potentiostat (Autolab PGSTAT302N) equipment and rotating disk electrode (RDE) measurement. Platinum mesh electrode and saturated calomel electrode (SCE) were used as counter electrodes and reference electrodes, respectively. As a working electrode, glassy-carbon RDE was used, on which catalyst ink was raised to a target of 10 μg_Pt_ cm^-2^. To make catalyst ink, 3.3 mg of catalyst powder and 3.4 µL of Nafion® ionomer was dispersed in 3.120 mL of DI water and n-propanol (70:30 volumetric ratio). Sonication of the mixed solution was evenly carried out for 1 hour of sonication, and 10 μL was added to a glassy carbon disk using a micropipette. The solvent then evaporated in the ambient atmosphere with the electrode rotating at 600 rpm.

For electrochemical performance evaluation, linear sweep voltammetry (LSV) was used under the conditions of scan rate of 20 mV s^-1^, rotation speed of 1,600 rpm, operation voltage of 0.05 to 1.1 V_RHE_, under O­_2_-sat. 0.1 M HClO_4_. The ORR polarization curve went through a process of calibration by removing background iR drop caused by electrolyte resistance from LSV measurement. Electrolyte resistance was measured by electrochemical impedance spectroscopy (EIS) analysis at 0.9 V_RHE_. Cyclic voltammetry was performed under scan rate of 50 mV s^-1^ without rotation in Ar-sat. 0.1 M HClO_4_ from 0.05 to 1.2 V_RHE_. For CO stripping voltammetry, CO gas was purged for 10 min and the purged with Ar gas for another 10 min while rotating the electrode at 400 rpm. The ECSA calculation in half-cell was performed through area integration of CO adsorption peak extruding background CV curve at potential window between 0.05 to 1.0 V_RHE_ at a scan rate of 20 mV s^-1^ without rotation.

The Accelerated Stress Test (AST) was conducted by holding 0.6 V_RHE_ and 0.95 V_RHE_ for 3 s each in Ar-Sat. 0.1 M HClO_4_ electrolyte, an actual MEA driving condition[3]. At this time, the voltage was maintained for 3 seconds each and then proceeded and changed into next step within 0.05 second, in which can be called as square wave cycling method (SWC). After 30,000 AST cycles, LSV, CV, EIS measurement, and CO stripping were performed. Metal dissolution during AST was quantified by ICP-MS (iCAP RQ) with the electrolyte volume of 70 mL.

Rotating ring-disk electrode (RRDE) measurement was conducted to confirm electron transfer number (n) and hydrogen peroxide (H_2_O_2_) yield. Similar with ink preparation for RDE measurement, 10 μL of catalyst ink was deposited onto a glassy carbon electrode (GCE) of 0.248 cm^2^ (AFE7R9GCPT, Pine Research) and dried at room temperature while electrode rotating at 600 rpm. Pt loading was set to be 20 μg_Pt_ cm^-2^. Ring electrode of 0.187 cm^-2^ was held at 1.2 V_RHE_ during measurement. Electron transfer number (n) and H_2_O_2_ yield could be acquired using following formula:

$$\text{n = 4 * }\frac{\text{I}_{\text{Disk}}}{\text{I}_{\text{Disk}}\text{+}\text{I}_{\text{Ring}}\text{/N}}$$

$$\text{\% }\text{H}_{\text{2}}\text{O}_{\text{2}}\text{ = 200 * }\frac{\text{I}_{\text{Ring}}\text{/N}}{\text{I}_{\text{Disk}}\text{+}\text{I}_{\text{Ring}}\text{/N}}$$

where N is the ring current collection efficiency determined to be 0.37.

For post-mortem analysis, the catalyst was uniformly spray-coated onto a 5 cm^2^ carbon paper (TGP-H-060, NARA Cell-Tech Corporation) at a Pt loading of 0.10 mg_Pt_ cm^-2^. A 1 cm^2^ section was cut from the coated carbon paper and used as the working electrode. A Pt mesh and a saturated calomel electrode (SCE) were used as the counter and reference electrodes, respectively, with 0.1 M HClO_4_ as the electrolyte. This configuration was adopted to mimic the catalyst layer environment in membrane electrode assemblies while allowing post-mortem structural and spectroscopic characterization. Prior to durability testing, the electrode was activated by cycling between 0 and 1.2 V_RHE_ at 50 mV s^-1^ for 100 cycles under Ar purging. Accelerated stress tests (ASTs) were then performed for 10,000 cycles using a square-wave protocol with 3 s holds at 0.60 and 0.95 V_RHE_.

*Single-Cell Electrochemical Characterizations*

The performance of PtCu@Pt NS/C was evaluated in a single cell by fabricating membrane-electrode assembly (MEA). As an anode catalyst, 60 wt.% Pt/C purchased from CNL Energy was used. The cathode catalyst ink was prepared by dispersing 3.5 mg of catalyst powder into the mixture of 13.6 µL of Nafion® solution in 4 mL of DI water and n-propanol. The prepared catalyst inks were sonicated for 30 minutes and uniformly sprayed onto the Nafion® 212 membrane using a spray coater (NadeTech). The catalyst loading was 0.05 mg_Pt_ cm^-2^ for the cathode and 0.10 mg_Pt_ cm^-2^ for the anode, respectively[4-7].

The prepared MEAs were assembled with a PTFE-coated gas diffusion layer (SGL SIGRACET®, 36BB, 280 µm) and graphite bipolar plates with serpentine flow fields without hot press. MEAs were pre-activated by circulating 0.5 M H_2_SO_4_ solution for 24 h and rinsed with DI[8-9]. The single-cell was activated using a protocol of a gradual voltage drop to 0.4 V followed by a 4-minute hold. Hydrogen and oxygen (CO-free) were supplied to the anode and cathode at the flow rate of 200 and 600 sccm without back pressure. Relative humidity of hydrogen and oxygen were maintained at 100 % while cell temperature was controlled at 65 ^o^C. The i-V polarization curve was measured by increasing the current stepwise and holding at each step for 1 minute until the cell voltage was saturated. For the accelerated stress test (AST), fully humidified hydrogen and nitrogen were supplied for the anode side and cathode side at the flow rate of 200 sccm each, operated under 65 ^o^C, ambient pressure, and conducted square-wave cycling (SWC) between 0.6 V and 0.95 V while holding 3 seconds each. After 30,000 AST cycles, i-V polarization curves were measured using the same protocol[10].

The catalyst layer proton transport resistance (R_ion_) was quantified using potentiostatic electrochemical impedance spectroscopy (EIS) under H_2_-N_2_ conditions based on a transmission line model [11-13]. EIS spectra were recorded at 0.4 V over a frequency range from 100 kHz to 0.1 Hz with an AC perturbation amplitude of V_a_ = 10 mV. R_ion_ is calculated via the following relationship:

R_ion_ = 3 * (Z’_extrapolated_ – R_ohm_)

Where R_ohm_ represents the ohmic resistance determined by the high-frequency real-axis intercept in the Nyquist plot, and Z’_extrapolated_ is the real-axis intercept obtained by extrapolating the linear transmission-line region to the real axis (Z_Re_).

**Figure S1.** (a) High-magnification top-view HAADF-STEM image, (b) Side view bright-field TEM image, (c) selected area electron diffraction (SAED) pattern ring, and (d) enlarged top-view HAADF-STEM image of PtCu NS/C. Inset shows corresponding fast Fourier transformed (FFT) pattern.

**Figure S2.** (a) Low magnification and (b) high magnification bright-field TEM image of Pt/C used as the reference in this study. The inset in (b) shows the particle size histogram.

**Figure S3.** Low magnification bright-field TEM image, and EDS elemental mapping image with corresponding atomic ratio (green for Pt intensity, and red for Cu intensity) of (a-b) PtCu@Pt NS/C, and (c-d) PtCu NS/C.

**Figure S4.** High magnification top-view and side-view EDS elemental mapping image with corresponding atomic ratio (green for Pt intensity, and red for Cu intensity) of (a-b) PtCu@Pt NS/C, and (c-d) PtCu NS/C.

**Figure S5.** TEM images of (a) additional Pt skin on PtCu NS/C, (b) acid-treated/annealed PtCu NS/C, and (c) Pt-rich PtCu NS/C. (d) XRD spectra of additional Pt skin on PtCu NS/C, acid-treated/annealed PtCu NS/C, Pt-rich PtCu NS/C, and PtCu@Pt NS/C.

**Table S1.** Dependence of Pt content and atomic ratio on the stoichiometric amount of Pt precursor for galvanic replacement reaction.

| **Sample** | **Assumed Cu Replacement in the Outmost Layer(s)** | **XRD** | **Theoretically**  **Expected Pt:Cu Atomic Ratio^a^** | **ICP-OES** | |
| --- | --- | --- | --- | --- | --- |
|  |  | **(111) peak** |  | **Pt wt.%** | **Pt:Cu Atomic Ratio** |
| **PtCu NS/C** | As-Synthesized | 41.4^o^ | 50:50 | 16.4 % | 45:55 |
| Condition 1 | 25 % | 41.1^o^ | 55:45 | 17.8 % | 55:45 |
| Condition 2 | 50 % | 41.0^o^ | 60:40 | 19.1 % | 58:42 |
| Condition 3  (**PtCu@Pt NS/C**) | 100 % | 40.9^o^ | 70:30 | 22.0 % | 68:32 |
| Condition 4 | Excess (200 %) | 40.9^o^ | 90:10 | 22.6 % | 70:30 |

*^a^ Theoretical Pt:Cu atomic ratios were calculated using an 5-atomic-layer nanosheet model with an initial Pt: Cu ratio of 50:50, distinct from the experimentally measured ratio of 45:55, to illustrate the expected compositional change upon near-surface Cu replacement.*

**Figure S6.** XRD patterns of PtCu NS/C and PtCu@Pt NS/C series with varying Pt-skin coverage.

**Figure S7.** Low magnification and high-magnification bright-field TEM images of PtCu@Pt NS/C series with varying Pt skin coverage, targeting (a-b) 25 %, (c-d) 50 %, and (e-f) 200 % of surface Cu to be exchanged.

**Figure S8.** Time-resolved ICP-OES data for Pt and Cu in solution. (a) with Pt precursor and (b) without Pt precursor.

**Figure S9.** (a) High magnification STEM image and (b) corresponding EDS line scanning profile of PtCu NS/C.

**Figure S10.** HAADF-STEM images and corresponding intensity profiles of eight individual nanosheets of PtCu NS/C.

**Figure S11.** High magnification STEM image of PtCu@Pt NS/C.

**Figure S12.** HAADF-STEM images and corresponding intensity profiles of eight individual nanosheets of PtCu@Pt NS/C.

**Figure S13.** Strain distribution (ε_xy_) of PtCu@Pt NS/C.

**Figure S14.** Strain distribution (ε_xy_) of PtCu NS/C.

**Figure S15.** (a) Survey and (b) high-resolution C 1s XPS spectra of PtCu@Pt NS/C, PtCu NS/C, and Pt/C.

**Figure S16.** High-resolution XPS spectra of PtCu@Pt NS/C, PtCu NS/C, and Pt/C: (a) Pt 4f and (b) Cu 2p.

**Table S2.** Main peak and peak assignment (%) change for the Pt 4f and Cu 2p XPS envelopes for PtCu@Pt NS/C, PtCu NS/C, and Pt/C.

| **Sample** | **Pt 4f** | | **Cu 2p** | |
| --- | --- | --- | --- | --- |
|  | **Main Peak** | **Pt(0) : Pt(II) : Pt(IV)** | **Main Peak** | **Cu(0) : Cu(II)** |
| **PtCu@Pt NS/C** | 71.7 eV | 56 : 27 : 17 | 932.3 eV | 77 : 23 |
| **PtCu NS/C** | 71.6 eV | 52 : 28 : 20 | 932.3 eV | 57 : 43 |
| **Pt/C** | 71.9 eV | 52 : 34 : 14 | - | - |

**Figure S17.** Pt L_3_-edge (a) XANES spectra, and (b) enlarged region of the whiteline intensity of PtCu@Pt NS/C, PtCu NS/C, commercial Pt/C, PtO_2_, and Pt foil.

**Figure S18.** Cu K-edge XANES spectra of PtCu@Pt NS/C, PtCu NS/C, CuO, Cu_2_O, and Cu foil. (b) Cu K-edge XANES spectra of PtCu NS/C. The dotted line is a linear fitting result of Cu species.

**Figure S19.** k^3^-weighted EXAFS spectra of PtCu@Pt NS/C, PtCu NS/C, commercial Pt/C, PtO_2_, and Pt foil.

**Figure S20.** EXAFS fitting result on k-space and R-space of (a-b) PtCu@Pt NS/C, and (c-d) PtCu NS/C. The scattered dots are experimental data and solid lines are fitting results.

**Table S3.** EXAFS fitting parameters for PtCu@Pt NS/C, PtCu NS/C, and Pt foil.

| **Sample** | **Pt:Cu** | **Path** | **C.N.** | **R (Å)** | **σ (Å^2^) * 10^-3^** | **Δ E_0_ (eV)** | **R-factor** |
| --- | --- | --- | --- | --- | --- | --- | --- |
| **PtCu@Pt NS/C** | 68:32 | Pt-Cu | 5.72 ± 0.61 | 2.65 ± 0.002 | 17.8 ± 1.6 | 1.14 ± 0.40 | 0.0083 |
|  |  | Pt-Pt | 5.57 ± 0.22 | 2.68 ± 0.004 | 3.7 ± 0.4 |  |  |
| **PtCu NS/C** | 47:53 | Pt-Cu | 5.77 ± 0.37 | 2.64 ± 0.004 | 10.5 ± 0.5 | 8.42 ± 0.53 | 0.0149 |
|  |  | Pt-Pt | 2.63 ± 0.33 | 2.71 ± 0.007 | 4.0 ± 0.8 |  |  |
| **Pt foil** | - | Pt-Pt 1 | **12** | 2.76 ± 0.003 | 4.5 ± 0.1 | 7.33 ± 0.45 | 0.0023 |
|  |  | Pt-Pt 2 | **6** | 3.90 ± 0.002 | 10.5 ± 3.3 |  |  |

* The value of S_0_^2^ for the Pt foil was estimated as 0.83. The EXAFS fitting range for each sample corresponds to the R range (1.2 – 3.0 Å) and the k-range (3.0 – 11.0 Å). Bold numbers indicate the fixed coordination number of each path to obtain the best EXAFS fitting based on FEFF calculations.

**Scheme S1.** Schematic illustration of the relationship between the oxidized state of Cu and Cu exposure.

**Figure S21.** k^3^-weighted wavelet transformed Cu K-edge EXAFS spectra of (a) Cu foil, and (b) Cu_2_O.

**Figure S22.** k^3^-weighted wavelet transformed Pt L_3_-edge EXAFS spectra of (a) PtCu@Pt NS/C, (b) PtCu NS/C, (c) commercial Pt/C, (d) PtO_2_, and (e) Pt foil.

**Figure S23.** Cyclic voltammetry of PtCu@Pt NS/C, PtCu NS/C, and Pt/C.

**Figure S24.** Change on CO stripping peak of (a-b) PtCu@Pt NS/C, (c-d) PtCu NS/C, and (e-f) commercial Pt/C before and after 30,000 AST cycles.

**Figure S25.** Tafel slope of PtCu@Pt NS/C, PtCu NS/C, and Pt/C.

**Figure S26.** Ring current and disk current acquired at different rotating speed from RRDE measurement for (a) PtCu@Pt NS/C, (b) PtCu NS/C, and (c) Pt/C.

**Figure S27.** Calculated H_2_O_2_ yield and electron transfer number of PtCu@Pt NS/C, PtCu NS/C, and Pt/C by RRDE measurement.

**Figure S28.** (a) ORR polarization curves and (b) CO stripping peak shift of PtCu NS/C before and after 30,000 AST cycles.

**Figure S29.** (a) ORR polarization curves and (b) CO stripping peak shift of Pt/C before and after 30,000 AST cycles.

**Figure S30.** (a) Mass activity, (b) ECSA, and (c) specific activity of PtCu@Pt NS/C, PtCu NS/C, and Pt/C before and after 30,000 AST cycles.

**Figure S31.** (a) Low magnification and (b) high magnification bright-field TEM image of commercial Pt/C after 30,000 AST cycles. The inset in (b) shows the particle size histogram.

**Figure S32.** Low magnification bright-field TEM image, and top view HAADF-STEM image of (a-b) PtCu@Pt NS/C, and (c-d) PtCu NS/C after 30,000 AST cycles.

**Figure S33.** High magnification top-view and side-view EDS elemental mapping image with corresponding atomic ratio (green for Pt intensity, and red for Cu intensity) of (a-b) PtCu@Pt NS/C, and (c-d) PtCu NS/C.

**Figure S34.** High-magnification side-view STEM image and corresponding EDS line scanning profile of (a-b) PtCu@Pt NS/C, and (c-d) PtCu NS/C after 30,000 AST cycles.

**Figure S35.** Metal dissolution from PtCu@Pt NS/C, PtCu NS/C, and Pt/C after 30,000 AST cycles in 0.1 M HClO_4_ electrolyte.

**Figure S36.** XRD peak shift of PtCu@Pt NS/C, PtCu NS/C, and Pt/C on carbon paper before and after 10,000 AST cycles.

**Figure S37.** (a) Survey and (b) high-resolution C 1s XPS spectra of PtCu@Pt NS/C, PtCu NS/C, and Pt/C on carbon paper.

**Figure S38.** High-resolution XPS spectra of PtCu@Pt NS/C, PtCu NS/C, and Pt/C on carbon paper: (a) Pt 4f and (b) Cu 2p.

**Table S4.** Main peak and peak assignment (%) change for the Pt 4f and Cu 2p XPS envelopes for PtCu@Pt NS/C, PtCu NS/C, and Pt/C on carbon paper.

| **Sample** | **Pt 4f** | | **Cu 2p** | |
| --- | --- | --- | --- | --- |
|  | **Main Peak** | **Pt(0) : Pt(II) : Pt(IV)** | **Main Peak** | **Cu(0) : Cu(II)** |
| **PtCu@Pt NS/C** | 71.7 eV | 53 : 39 : 8 | 932.5 eV | 64 : 36 |
| **PtCu NS/C** | 71.6 eV | 52 : 38 : 10 | 932.5 eV | 68 : 32 |
| **Pt/C** | 71.9 eV | 56 : 30 : 14 | - | - |

**Figure S39.** (a) Survey and (b) high-resolution C 1s XPS spectra of PtCu@Pt NS/C, PtCu NS/C, and Pt/C on carbon paper after 10,000 AST cycles.

**Figure S40.** High-resolution XPS spectra of PtCu@Pt NS/C, PtCu NS/C, and Pt/C after 10,000 AST cycles: (a) Pt 4f and (b) Cu 2p.

**Table S5.** Main peak and peak assignment (%) change for the Pt 4f and Cu 2p XPS envelopes for PtCu@Pt NS/C, PtCu NS/C, and Pt/C on carbon paper after 10,000 AST cycles.

| **Sample** | **Pt 4f** | | **Cu 2p** | |
| --- | --- | --- | --- | --- |
|  | **Main Peak** | **Pt(0) : Pt(II) : Pt(IV)** | **Main Peak** | **Cu(0) : Cu(II)** |
| **PtCu@Pt NS/C** | 71.7 eV | 56 : 33 : 11 | 932.5 eV | 78 : 22 |
| **PtCu NS/C** | 71.7 eV | 57 : 31 : 12 | 932.5 eV | 77 : 23 |
| **Pt/C** | 71.9 eV | 65 : 23 : 12 | - | - |

**Table S6.** Decrease in transition metal atomic fractions of Pt-transition metal alloy catalysts after ORR durability testing.

| Reported Catalyst | Pristine  atomic  ratio^a^ | AST Protocol^b^ | Cycles | Mass  Activity  [A mg_Pt_^-1^] | ECSA  [m^2^ g_Pt_^-1^] | Final atomic ratio  (TM loss^c^) | Ref. |
| --- | --- | --- | --- | --- | --- | --- | --- |
| PtCu@Pt NS/C | Pt_70_Cu_30_ | SWC  0.6 - 0.95 V_RHE_ | Pristine | 1.71 | 70 | Pt_75_Cu_25_  (-5 pp) | This Work |
|  |  |  | 30,000 | 1.41 | 61 |  |  |
| PtCu NS/C | Pt_41_Cu_59_ | SWC  0.6 - 0.95 V_RHE_ | Pristine | 2.23 | 74 | Pt_70_Cu_30_  (-29 pp) | This Work |
|  |  |  | 30,000 | 1.58 | 56 |  |  |
| L1_0_-Pt_2_CuGa/C | Pt_50_Cu_25_Ga_25_ | TWC  0.6 ~ 1.0 V_RHE_ | Pristine | 1.39 | 48.6 | Pt_60_Cu_20_Ga_20_  (-10 pp) | [14] |
|  |  |  | 30,000 | - | 37.0 |  |  |
| L1_1_-PtCu | Pt_52_Cu_48_ | TWC  0.6 ~ 1.0 V_RHE_ | Pristine | 0.82 | 66.2 | Pt_91_Cu_9_  (-39 pp) | [15] |
|  |  |  | 10,000 | 0.25 | 39.2 |  |  |
| L1_0_-PtCo/ZnNC | Pt_45_Co_40_Zn_15_ | SWC  0.6 - 0.95 V_RHE_ | Pristine | 1.76 | 69.8 | Pt_57_Co_34_Zn_9_  (-12 pp) | [16] |
|  |  |  | 30,000 | 1.12 | 66.8 |  |  |
| L1_0_-PtCo/C | Pt_49_Co_51_ | SWC  0.6 - 0.95 V_RHE_ | Pristine | 0.77 | 78.8 | Pt_72_Co_28_  (-23 pp) | [16] |
|  |  |  | 30,000 | 0.42 | 55.7 |  |  |
| PtFe@C-H20 | Pt_75_Fe_25_ | TWC  0.6 ~ 1.1 V_RHE_ | Pristine | 2.3 | 86 | Pt_83_Fe_17_  (-8 pp) | [17] |
|  |  |  | 20,000 | 1.1 | 52 |  |  |

*^a^ To methodological consistency, all pristine and final atomic ratios in this table are derived from STEM-EDS analyses.*

*^b^ Direct quantitative comparisons should be made with caution because catalyst compositions and AST protocols, including potential windows, cycling modes, and cycle numbers, differ among studies.*

*^c^ TM loss represents the decrease in the total atomic fraction of non-Pt transition metal(s) from the pristine to the post-AST state, expressed in percentage points (pp).*

**Figure S41.** i-V curves on H_2_-O_2_ 1 bar operation of (a) PtCu@Pt NS/C, (b) PtCu NS/C and (c) Pt/C before and after 30,000 AST cycles.

**Figure S42.** Voltage loss at 1.2 A cm^-2^ after 30,000 AST cycles of PtCu@Pt NS/C, PtCu NS/C, and Pt/C.

**Figure S43.** i-V curves on H_2_-Air 1 bar operation of (a) PtCu@Pt NS/C, (b) PtCu NS/C and (c) Pt/C before and after 30,000 AST cycles.

**Table S7.** Electrochemical durability comparison of PGM-based nanosheet catalysts for oxygen reduction reaction.

| Catalyst | Catalyst Morphology | | Half-cell Test | | | | Single-cell Test | | | | | Ref. |
| --- | --- | --- | --- | --- | --- | --- | --- | --- | --- | --- | --- | --- |
|  | Lateral Size  [nm] | Thick  ness  [nm] | AST Protocol | Cycles | Mass  Activity  [A mg_Pt_^-1^] | ECSA  [m^2^ g_Pt_^-1^] | Loading  [mg_Pt_ cm^-2^] | AST Protocol | Cycles | Voltage at  0.2 A cm^-2^  [mV] | Voltage at  1.2 A cm^-2^  [mV] |  |
| PtCu@Pt  NS/C | 15 | 1 | SWC  0.6 – 0.95 V_RHE_ | Pristine | 1.71 | 70 | 0.05 | SWC  0.6 – 0.95 V_RHE_ | Pristine | 730 | 480 | This Work |
|  |  |  |  | 30,000 | 1.41 | 61 |  |  | 30,000 | 726 | 457 |  |
| PtCu  NS/C | 15 | 1 | SWC  0.6 – 0.95 V_RHE_ | Pristine | 2.23 | 74 | 0.05 | SWC  0.6 – 0.95 V_RHE_ | Pristine | 734 | 508 | This Work |
|  |  |  |  | 30,000 | 1.58 | 56 |  |  | 30,000 | 693 | 386 |  |
| PdRh-Pt/C | 8.25 | 2.59 | - | Pristine | 0.7943 | - | - | - | - | - | - | [18] |
|  |  |  |  | - | - | - |  |  | - | - | - |  |
| Pt_0.88_Co_0.12_-NS/C | 841 | 1.5 | SWC  0.6 – 1.0 V_RHE_ | Pristine | 0.468 | 102 | - | - | - | - | - | [19] |
|  |  |  |  | 5,000 | 0.220 | 71 |  |  | - | - | - |  |
| Pt_3_Pd_33_Cu_64_ NS/C | 500 | 1.22 | - | Pristine | 1.59 | 376 | - | - | - | - | - | [20] |
|  |  |  |  | - | - | - |  |  | - | - | - |  |
| Pt_3_Ni HU-NS/C | 260 | 2.5 | - | Pristine | 2.27 | 48.32 | - | - | - | - | - | [21] |
|  |  |  |  | - | - | - |  |  | - | - | - |  |
| Pt NS/C | 500 | 0.5 | SWC  0.6 – 1.0 V_RHE_ | Pristine | 0.42 | 124 | - | - | - | - | - | [22] |
|  |  |  |  | 50,000 | - | 93 |  |  | - | - | - |  |
| HW-Pd NS/C | 500 | 1.35 | TWC  0.6 ~ 1.1 V_RHE_ | Pristine | 0.26 | 81.2 | - | - | - | - | - | [23] |
|  |  |  |  | 30.000 | - | 73.1 |  |  | - | - | - |  |
| Pd NS/C | 500 | 1.35 | TWC  0.6 ~ 1.1 V_RHE_ | Pristine | 0.16 | 51.1 | - | - | - | - | - | [23] |
|  |  |  |  | 30,000 | - | 31.2 |  |  | - | - | - |  |
| E-Pt NS/C | 100 | 0.9 | TWC  0.6 ~ 1.1 V_RHE_ | Pristine | 2.07 | 70.2 | - | - | - | - | - | [24] |
|  |  |  |  | 30,000 | 1.71 | 56.5 |  |  | - | - | - |  |
| A-Pt NS/C | 100 | 0.9 | TWC  0.6 ~ 1.1 V_RHE_ | Pristine | 0.95 | 57.8 | - | - | - | - | - | [24] |
|  |  |  |  | 30,000 | 0.86 | 46.6 |  |  | - | - | - |  |
| PtFe NS/C | 2000 | 2 | - | Pristine | 0.57 | 36.4 | - | - | - | - | - | [25] |
|  |  |  |  | - | - | - |  |  | - | - | - |  |

** Direct quantitative comparisons should be made with caution because catalyst compositions and AST protocols, including potential windows, cycling modes, and cycle numbers, differ among studies.*

**Figure S44.** Low magnification bright-field TEM image, and EDS elemental mapping image and corresponding atomic ratio of (a-b) PtCu@Pt NS/C and (c-d) PtCu NS/C after 30,000 AST cycles.

**Figure S45.** SEM EDS line scan on PtCu@Pt NS/C and PtCu NS/C near cathode catalyst layer and Nafion® membrane before and after 30,000 AST cycles.

**Figure S46.** PEIS of (a) PtCu@Pt NS/C, (b) PtCu NS/C, and (c) Pt/C at 0.4 V before and after 30,000 AST cycles.

**Table S8.** Comparison of R_ohm_ and R_ion_ on PtCu@Pt NS/C, PtCu NS/C, and Pt/C.

| **Sample** | **AST Cycles** | **R_ohm_ (mΩ)** | **R_ion_ (mΩ)** |
| --- | --- | --- | --- |
| **PtCu@Pt NS/C** | Pristine | 16 | 10 |
|  | after 30 k | 16 | 11 |
| **PtCu NS/C** | Pristine | 16 | 13 |
|  | after 30 k | 16 | 18 |
| **Pt/C** | Pristine | 13 | 5 |
|  | after 30 k | 13 | 6 |

**Supporting References**

[1]	B. Ravel, M. Newville, *Journal of Synchrotron Radiation* **2005**, *12* (4), 537, https://doi.org/10.1107/S0909049505012719.

[2]	J. Roh, A. Cho, S. Kim, K.-S. Lee, J. Shin, J. S. Choi, J. Bak, S. Lee, D. Song, E.-J. Kim, C. Lee, Y. R. Uhm, Y.-H. Cho, J. W. Han, E. Cho, *ACS Catalysis* **2023**, *13* (14), 9427, https://doi.org/10.1021/acscatal.3c01136.

[3]	A. Marcu, G. Toth, R. J. Behm, *Fuel Cells* **2014**, *14* (3), 378, https://doi.org/10.1002/fuce.201300138.

[4]	T. Chen, F. Ning, J. Qi, G. Feng, Y. Wang, J. Song, T. Yang, X. Liu, L. Chen, D. Xia, *iScience* **2023**, *26* (1), 105890, https://doi.org/10.1016/j.isci.2022.105890.

[5]	P. Schneider, M. Batool, A. O. Godoy, R. Singh, D. Gerteisen, J. Jankovic, N. Zamel, *Journal of The Electrochemical Society* **2023**, *170* (2), 024506, https://doi.org/10.1149/1945-7111/acb8df.

[6]	G. Li, J. Bai, C. Liu, Z. Jin, M. Xiao, W. Xing, *Small* **2025**, *21* (1), 2407163, https://doi.org/10.1002/smll.202407163.

[7]	T. Chen, C. Qiu, X. Zhang, H. Wang, J. Song, K. Zhang, T. Yang, Y. Zuo, Y. Yang, C. Gao, W. Xiao, Z. Jiang, Y. Wang, Y. Xiang, D. Xia, *Journal of the American Chemical Society* **2024**, *146* (1), 1174, https://doi.org/10.1021/jacs.3c12649.

[8]	R. K. Ahluwalia, X. Wang, J. K. Peng, N. N. Kariuki, D. J. Myers, S. Rasouli, P. J. Ferreira, Z. Yang, A. Martinez-Bonastre, D. Fongalland, J. Sharman, *Journal of The Electrochemical Society* **2018**, *165* (6), F3316, https://doi.org/10.1149/2.0341806jes.

[9]	M. Gatalo, P. Jovanovič, U. Petek, M. Šala, V. S. Šelih, F. Ruiz-Zepeda, M. Bele, N. Hodnik, M. Gaberšček, *ACS Applied Energy Materials* **2019**, *2* (5), 3131, https://doi.org/10.1021/acsaem.8b02142.

[10]	S. Stariha, N. Macauley, B. T. Sneed, D. Langlois, K. L. More, R. Mukundan, R. L. Borup, *Journal of The Electrochemical Society* **2018**, *165* (7), F492, https://doi.org/10.1149/2.0881807jes.

[11]	J. Jung, Y.-H. Chung, H.-Y. Park, J. Han, H.-J. Kim, D. Henkensmeier, S. J. Yoo, J. Y. Kim, S. Y. Lee, K. H. Song, H. S. Park, J. H. Jang, *International Journal of Hydrogen Energy* **2018**, *43* (32), 15457, https://doi.org/10.1016/j.ijhydene.2018.06.093.

[12]	J. Kwon, S. Jo, K.-Y. Cho, K. Eom, *Journal of Power Sources* **2020**, *473*, 228587, https://doi.org/10.1016/j.jpowsour.2020.228587.

[13]	J. Lee, H. Salihi, J. Lee, H. Ju, *Energy* **2022**, *239*, 122294, https://doi.org/10.1016/j.energy.2021.122294.

[14]	X. Liu, Z. Zhao, J. Liang, S. Li, G. Lu, C. Priest, T. Wang, J. Han, G. Wu, X. Wang, Y. Huang, Q. Li, *Angewandte Chemie International Edition* **2023**, *62* (23), e202302134, https://doi.org/10.1002/anie.202302134.

[15]	M. Gong, D. Xiao, Z. Deng, R. Zhang, W. Xia, T. Zhao, X. Liu, T. Shen, Y. Hu, Y. Lu, X. Zhao, H. Xin, D. Wang, *Applied Catalysis B: Environmental* **2021**, *282*, 119617, https://doi.org/10.1016/j.apcatb.2020.119617.

[16]	H. Chang, J. H. Ryu, K. Lee, J. Roh, S. Lee, J. Bak, D. Shin, M. Kim, H. Yang, W. B. Lee, E. Cho, Advanced Energy Materials 2026, 16 (11), e05211, https://doi.org/10.1002/aenm.202505211.

[17]	M.-G. Kim, S. S. Chougule, S. Lee, Y. S. Park, J. Choi, K. H. Lim, A. S. Lee, H. S. Park, N. Jung, S. J. Yoo, Advanced Functional Materials 2026, 36 (45), e75620, https://doi.org/https://doi.org/10.1002/adfm.75620.

[18]	J. Jiang, J. Wu, C. Yang, S. Bo, J. Zhang, B. Li, Y. Zhang, Q. An, X. Chen, Q. Liu, W. Zhou, *Angewandte Chemie International Edition* **2025**, *64* (35), e202510188, https://doi.org/https://doi.org/10.1002/anie.202510188.

[19]	O. R. M. Metawea, S. Tominaka, A. Machida, W. Sugimoto, *ACS Applied Materials & Interfaces* **2026**, *18* (25), 35439, https://doi.org/10.1021/acsami.6c08962.

[20]	Y. Li, J. Cheng, Q. Wang, H. Wang, H. Hou, Y. Zhu, J. Sun, M. Shi, X. Zhang, *Catalysis Letters* **2025**, *155* (12), 398, https://doi.org/10.1007/s10562-025-05232-0.

[21]	H. C. Kim, R. K. Pramadewandaru, M. K. Kabiraz, G. A. B. Azizar, H. Wahidah, Y. Kim, S.-U. Lee, H.-J. Chae, S.-I. Choi, J. W. Hong, *ACS Catalysis* **2024**, *14* (6), 3756, https://doi.org/10.1021/acscatal.3c05806.

[22]	D. Takimoto, S. Toma, Y. Suda, T. Shirokura, Y. Tokura, K. Fukuda, M. Matsumoto, H. Imai, W. Sugimoto, *Nature Communications* **2023**, *14* (1), 19, https://doi.org/10.1038/s41467-022-35616-4.

[23]	L. Y. Zhang, C. X. Guo, H. Cao, S. Wang, Y. Ouyang, B. Xu, P. Guo, C. M. Li, *Chemical Engineering Journal* **2022**, *431*, 133237, https://doi.org/10.1016/j.cej.2021.133237.

[24]	Y. Feng, B. Huang, C. Yang, Q. Shao, X. Huang, *Advanced Functional Materials* **2019**, *29* (45), 1904429, https://doi.org/10.1002/adfm.201904429.

[25]	W. Chen, W. Gao, P. Tu, T. Robert, Y. Ma, H. Shan, X. Gu, W. Shang, P. Tao, C. Song, T. Deng, H. Zhu, X. Pan, H. Yang, J. Wu, *Nano Letters* **2018**, *18* (9), 5905, https://doi.org/10.1021/acs.nanolett.8b02606.
